# Difference in marginal bone loss around implants between short implant-supported partial fixed prostheses with and without cantilever: a retrospective clinical study

**DOI:** 10.1186/s40729-023-00515-w

**Published:** 2023-12-01

**Authors:** Josef Al-Kilani, Sedef Al-Kilani, Bruno Ramos Chrcanovic

**Affiliations:** 1 Faculty of Odontology, Malmö University, Malmö, Sweden; 2 Department of Prosthodontics, Faculty of Odontology, Malmö University, Malmö, Sweden

**Keywords:** Dental implants, Fixed dental prosthesis, Cantilever extension, Marginal bone loss, Retrospective clinical study

## Abstract

**Purpose:**

To investigate the influence of cantilever prosthetic arm on the marginal bone loss (MBL) over time around dental implants supporting short fixed partial dentures (FPDs), in a record-based retrospective study.

**Methods:**

All cases of 3-unit implant-supported FPDs, supported by 2–3 implants, from the database of cases treated at one specialist clinic were considered for inclusion. Only implants with a minimum of 36 months of radiological follow-up were considered. Univariate linear regression models were used to compare MBL over time between 12 clinical covariates, after which a linear mixed-effects model was built.

**Results:**

One-hundred-thirty-nine patients (64 men, 75 women) with 164 3-unit implant-supported FPDs (333 implants supporting non-cantilevered FPDs, 94 supporting cantilevered FPDs) were included in the study. The patients were followed up clinically and radiographically for a mean of 154.1 ± 78.0 (min–max, 37.3–364.6) and 132.9 ± 77.3 months (min–max, 36.8–329.9), respectively. The total number of marginal bone level double measurements (mesial and distal sides of each implant) was 2909. FPDs with cantilever presented an estimated greater MBL over time compared to FPDs without cantilever. Bruxism, sex (women), implant (modified) surface, and (poor) bone quality were also associated with higher MBL over time.

**Conclusions:**

The use of a cantilever extension is suggested to negatively affect the bone marginal level over time around implants supporting 3-unit FPDs. Due to the small difference of the estimated MBL over long periods of follow-up between the groups, it is a matter of debate if the observed negative effect may be of clinical significance.

**Graphical Abstract:**

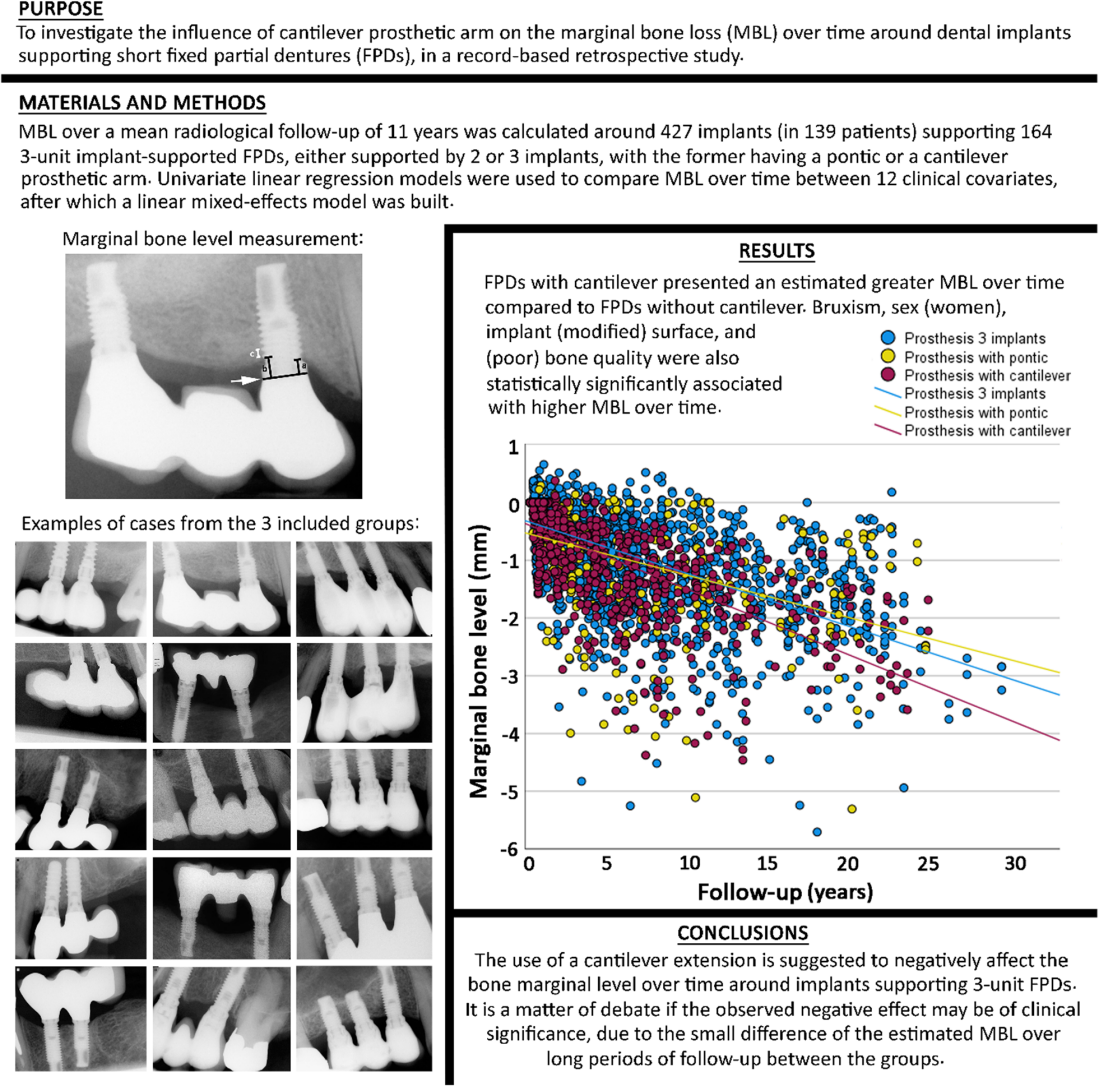

## Introduction

Cantilevers are used to extend implant-supported fixed prosthesis beyond the region directly supported by teeth or implants. In the case of rehabilitation with dental implants, the advantage of the extension of dental prostheses with cantilevers include the reduction in the cost of the rehabilitation, allowing for more prosthetic units without the need of an extra supporting implant. Moreover, cantilevers can also be used to avoid grafting in both the maxillary sinus and posterior mandible [1]. With the same purpose, cantilevers can also be directed anteriorly.

However, it has been suggested that the use of cantilever in implant-supported restorations may increase the risk of mechanical complications, prosthesis failure, and even implant failure [2, 3]. Cantilever may also be associated with excessive stress in the marginal bone around implants. Finite-element analysis (FEA) and photoelastic studies have suggested that stress values along the cervical region in the alveolar bone around implants increased with an increased cantilever length [4–7]. It is possible that excessive pressure applied to the bone in areas of high strain concentrations could cause osseous micro-fractures. In addition, excessive force concentrations may cause bone loss around implants [8, 9].

Some clinical studies also looked into the relationship between bone loss around dental implants and cantilever, with conflicting results. Studies with follow-ups of about 5 years failed to demonstrate that the presence of cantilever extensions in a fixed partial denture (FPD) had an effect on peri-implant bone loss [10, 11], the same conclusion reached in 3-year clinical that investigated a single implant to support a two-unit cantilever fixed dental prosthesis [12]. On the other hand, the marginal bone level around implants supporting fixed prostheses was observed to be negatively affected by the presence of a cantilever, in a 3-year retrospective clinical study [13]. The authors of the study recommended that short and/or narrow implants should be preferred over cantilever extensions in cases of limiting anatomic conditions. Another retrospective study, with a mean follow-up of 51 months, observed that the length of the cantilever arm was positively correlated bone loss [1]. Therefore, a general consensus still does not exist.

The aim of the present retrospective study was to further investigate the influence of the cantilever prosthetic arm on the marginal bone loss (MBL) over time around dental implants supporting short partial fixed prostheses, in a long-term follow-up period.

## Materials and methods

### Objective

The aim of the present retrospective study was to investigate the influence of cantilever prosthetic arm on the marginal bone loss (MBL) around dental implants supporting short partial fixed prostheses.

### Hypothesis

The null hypothesis of the present study was that there will be no significant difference in MBL between 3-unit implant-supported partial fixed prostheses with and without cantilever prosthetic arm, against the alternative hypothesis of a difference.

### Materials

This retrospective study included patients treated with dental implants during the period 1980–2018 at one specialist clinic (Clinic for Prosthodontics, Centre of Dental Specialist Care, Malmö, Sweden). This study was based on data collection from patients’ dental records. The implants were placed by specialist dentists in oral surgery, and dentists performing the prosthetic treatment were specialists in prosthodontics.

The study was approved by the regional Ethical Committee, Lund, Sweden (Dnr 2014/598; Dnr 2015/72). The present retrospective study followed the STROBE guidelines for observational studies [14] and was registered at https://clinicaltrials.gov under the registration number NCT02369562. The investigation was conducted according to the principles embodied in the Helsinki Declaration of 1964 for biomedical research involving human subjects, as amended in 2013 [15].

### Definitions

A cantilever prosthetic arm was defined as a pontic which is retained and supported only on one side by the other prosthetic units which are supported by implants.

MBL was defined as loss, in an apical direction, of alveolar bone marginally adjacent to the dental implant, in relation to the marginal bone level initially detected after the implant was surgically placed [16].

For this study, patients smoking a minimum of one cigarette per day (an everyday smoker [17]) were classified as smokers, established at the clinical appointment of the patient when the anamnesis was performed.

The diagnosis of bruxism was established in a previous study [18], in which the patients of the aforementioned database (which the present cohort group of patients was selected from, according to the inclusion/exclusion criteria) suspected to be bruxers were called back for one clinical appointment to get the minimum information to diagnose the patients as ‘probable bruxers’ (self-report/anamnesis + clinical examination).

As the standard protocol in the clinic, the patients’ dental hygiene was followed up by a dental hygienist within 6 months after the final implant-supported/retained restoration. Each patient then attended a dental hygiene recall program based on individual needs.

### Inclusion and exclusion criteria

**Fig. 1:**
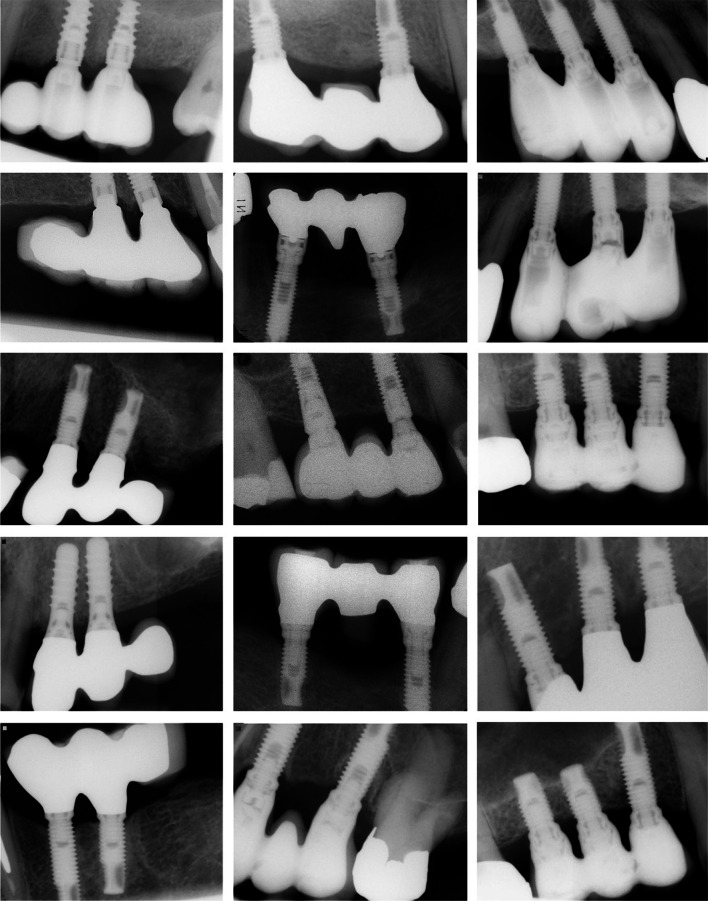
Examples of radiographs of cases included in the present study: 3-unit prosthesis supported by two implants with a cantilever (left column), 3-unit prosthesis supported by two implants with a pontic (middle column), and 3-unit prosthesis supported by three implants (right column)

Only implant-supported FPDs with three prosthetic units, either supported by two or three implants, were considered for inclusion. FPDs supported by two implants could had either a prosthetic pontic replacing a tooth between the implants, or a prosthetic arm cantilever (Fig. 1). Only implants not lost and with baseline radiographs taken within 12 months after implant placement and with a minimum of 36 months of radiological follow-up were considered for the analysis of MBL. Negative values of MBL corresponded to bone loss.

Patients with all modern types of threaded implants with cylindrical or conical design were included. Zygomatic implants were not included in the study, as well as implants detected in radiographies, but without basic information registered in the patients’ records.

Patients were excluded if they had history of periodontitis and/or were treated for periodontal disease. It is important to take note that as standard, all patients receiving implants at the Specialist Clinic for Prosthodontics were periodontally healthy at the time of implant installation. Patients with either a history or with signs of periodontal disease were treated at the Specialist Clinic for Periodontology, where they later could or not receive dental implants, according to individual needs/indications. These patients were not included in the present study.

### Data collection

The data were directly entered into a SPSS file (SPSS software, version 28, SPSS Inc., Chicago, IL, USA) as the dental records of the patients were being read, and it consisted of several implant-, site-, and patient-related factors. The following data were collected from the patients’ dental records:

- Implant-related factors: implant diameter (three groups: < 3.75, 3.75, and > 3.75 mm), system, and implant surface (turned/machined, modified);
- Site-related factors: implant region, implant jaw location (maxilla/mandible), anterior or posterior location of the implant (sites from right canine to left canine teeth were considered anterior location), bone quantity and quality of the implant site at the day of the implant installation, according to a classification [19];
- Surgery-related factors: open or flapless surgery, immediate installation in extraction socket or in healed site;
- Prosthetic-related factors: prosthesis fixation (screwed, cemented);
- Patient-related factors: patient’s sex, age of the patient at the implant insertion surgery, diabetes, and behavioral history (bruxism, smoking);
- Time-related factors: implant and prosthesis installation date, clinical and radiological follow-up time.

### Evaluation of radiographs

Reproducible intra-oral periapical radiographs were used. When there were no available digital radiographs from the baseline appointment, the analogue periapical radiographs were scanned at 1200 dpi (Epson Perfection V800 Photo Color Scanner; Nagano, Japan).

**Fig. 2:**
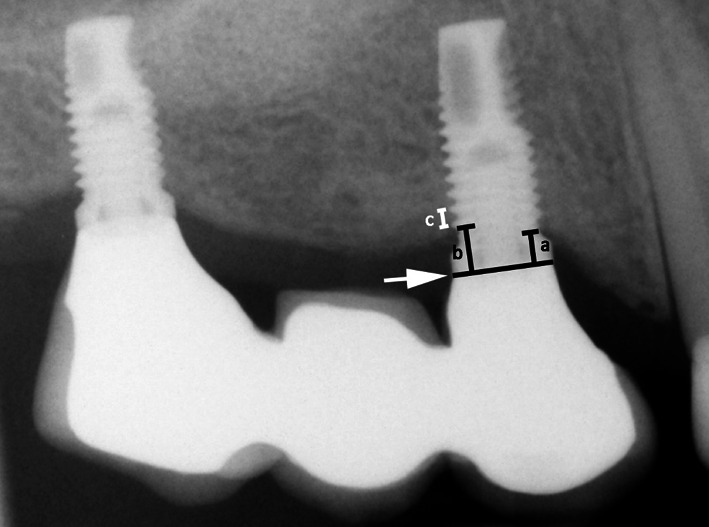
Measurement of the distance from the implant-abutment junction (black line indicated by the white arrow) to the first visible bone-to-implant contact, on both mesial (**a**) and distal (**b**) sides on periapical radiographs. Calibration was based on the inter-thread distance of the implants (**c**)

MBL was measured after calibration based on the inter-thread distance of the implants. Measurements were taken from the implant-abutment junction to the marginal bone level, at both mesial and distal sides of each implant, and then the mean value of these two measurements was considered (Fig. 2). MBL was calculated by comparing bone-to-implant contact levels to the radiographic baseline examination. The Image J software (National Institute of Health, Bethesda, USA) was used for all measurements.

The sets of radiographs for every patient were codified and the authors who performed the radiological measurements (S.A.K, J.A.K.) were blinded to the patients’ identification.

### Calibration

An initial calibration concerning MBL was performed between the authors. The process was done for 10 random samples from the cohort group, and verified after the measurement of each sample. At the end of the process the measurements from the different individuals were considered enough approximate from each other, with agreement between examiners set at > 90% of the distance in millimeters.

### Sample size calculation

A calculation of the sample size was not conducted. The reason is that the database from which the eligible cases for the present study were originated had a certain number of patients and dental implants, namely, approximately 2800 and 11,000, respectively, and it would not possible to recruit more cases, as the database already included all patients treated with dental implants during the aforementioned period in the specialist clinic.

Instead, all the 3-unit implant-supported FPDs were initially considered eligible for inclusion, to get the maximum number of cases available, namely, the largest sample size possible from this database, provided that these cases would fulfill the inclusion criteria, i.e., baseline radiographs taken within 12 months after implant placement and with a minimum of 36 months of radiological follow-up.

### Statistical analyses

The mean, standard deviation, and percentages were presented as descriptive statistics. Kolmogorov–Smirnov test was performed to evaluate the normal distribution of the variables, and Levene’s test evaluated homoscedasticity. The performed tests for two independent groups were Student’s *t* test or Mann–Whitney test, one way ANOVA or Kruskal–Wallis test for three independent groups, and paired-samples *t* test or Wilcoxon signed-rank test for two dependent groups, depending on the normality. Pearson’s Chi-squared test or Fisher’s exact test was used in the analysis of contingency tables of categorical data of independent groups. Correlation and linear regression were performed to check the relationships between MBL and time of follow-up.

Univariate linear regression models were used to compare MBL over time between clinical covariates. The estimation of MBL over time (dependent variable) was expressed in a single linear regression equation, for each of the categories of each independent variable (smoking, diabetes, bruxism, sex, age, jaw, jaw region, tooth region, implant diameter, implant surface, prosthesis type, prosthesis fixation). For the present study, the linear regression equation was expressed as

$$y = b + ax,$$

where ‘*y*’ is the estimated MBL over time. ‘*b*’ is the estimated intercept at the y-axle in the scatter plot. ‘*a*’ is the estimated MBL per every 1 month of follow-up. ‘*x*’ is the number of months of follow-up.

Thus, if one would like to estimate the MBL of a certain category of a certain variable at, for example, 100 months of follow-up, ‘*x*’ is replaced by the value of 100 in the equation given for that particular category and variable.

To verify multicollinearity, a correlation matrix of all of the predictor variables was scanned, to see whether there were some high correlations among the predictors. Collinearity statistics obtaining variance inflation factor (VIF) and tolerance statistic were also performed to detect more subtle forms of multicollinearity. A linear mixed-effects model was built with all variables that were moderately associated (*p* < 0.10) with MBL in the univariate linear regression models. Mixed-effects model was used to take into consideration that some patients had more than one implant-supported prostheses, as multiple observations within an individual are not independent of each other. Multiple testing corrections for *p* values were performed by the Bonferroni adjustment.

The degree of statistical significance was considered *p* < 0.05. Data were statistically analyzed using the Statistical Package for the Social Sciences (SPSS) version 28 software (SPSS Inc., Chicago, IL, USA).

## Results

There were 139 patients (64 men, 75 women) with 164 3-unit implant-supported FPDs (99 prostheses supported by 3 implants, 19 prostheses supported by two implants with a pontic, and 46 prostheses supported by two implants with a cantilever) fulfilling the inclusion criteria of a baseline radiograph taken within 12 months after implant placement and a minimum of 36 months of radiological follow-up. The FPDs were supported by 427 implants, all of which were installed with an open flap approach and in healed sites. Most of the implants of the study were Nobel Biocare implants (Göteborg, Sweden), totaling 368 implants (259 turned/machined and 109 TiUnite implants).

The mean age (± SD) of the 139 patients was 58.7 ± 13.7 years (min–max, 15.6–84.0) on the day of implant placement. The patients were followed up clinically for a mean (± SD) of 154.1 ± 78.0 months (min–max, 37.3–364.6), and radiographically for a mean (± SD) of 132.9 ± 77.3 months (min–max, 36.8–329.9).

Table 1 shows the descriptive data of the cases included in the study, separated by group. The variable of patient’s age was divided into three categories each, based on the 33.3 and 66.7 percentiles of sample distribution, to generate groups of more balanced sample sizes.

**Table 1:** Descriptive data of the implants included in the study, separated by group. The statistical unit is the implant

Factor	Prostheses without cantilever (%)	Prostheses with cantilever (%)	*p* value
Patients/Implants (n)	102^a^/333	43^a^/94	
Follow-up (months) (mean ± SD, min–max)			
Clinical	154.6 ± 77.5 (37.3–364.6)	152.4 ± 80.1 (56.7–315.4)	0.645^b^
Radiological	131.1 ± 77.4 (36.8–329.9)	139.0 ± 77.0 (38.0–300.6)	0.202^b^
Age (years)			
Mean ± SD	58.8 ± 14.1	58.4 ± 12.3	0.486^b^
< 56	101 (30.4)	32 (33.7)	
56.0–65.9	116 (34.8)	33 (34.7)	0.780^c,d^
≥ 66	116 (34.8)	30 (31.6)	
Sex			
Male	16 (43.8)	45 (47.4)	0.542^c,d^
Female	187 (56.2)	50 (52.6)	
Jaw			
Maxilla	107 (32.1)	50 (52.6)	< 0.001^c,d^
Mandible	226 (67.9)	45 (47.4)	
Jaw position			
Anterior	21 (6.3)	3 (3.2)	0.239^c,d^
Posterior	312 (93.7)	92 (96.8)	
Implant surface			
Turned	214 (64.3)	45 (47.4)	0.003^c,d^
Modified	119 (35.7)	50 (52.6)	
Implant diameter			
< 3.75 mm	18 (5.4)	9 (9.5)	
3.75 mm	287 (86.2)	67 (70.5)	0.001^c,d^
> 3.75 mm	28 (8.4)	19 (20.0)	
Prosthesis fixation^c^			
Cemented	2 (0.6)	2 (2.1)	0.219^c,e^
Screwed	326 (99.4)	93 (97.9)	
Bone quantity			
A–B	222 (66.7)	66 (69.5)	0.607^c,d^
C–D–E	111 (33.3)	29 (30.5)	
Bone quality			
1–2	144 (43.2)	36 (37.9)	0.352^c,d^
3–4	189 (56.8)	59 (62.1)	
Smoking^f^			
No	210 (80.5)	55 (71.4)	0.091^c,d^
Yes^g^	51 (19.5)	22 (28.6)	
Bruxism^f^			
No	229 (86.7)	65 (86.7)	0.986^c,d^
Yes	35 (13.3)	10 (13.3)	
Diabetes^f^			
No	235 (78.9)	63 (65.7)	0.078^c,d^
Yes	23 (21.1)	12 (34.3)	

*SD* standard deviation

^a^The total number of patients of the study was 139, but some patients had more than one prosthesis, sometimes prostheses with and prostheses without cantilever. That is why the total number of patients in both groups here artificially amounts to “145”

^b^Mann–Whitney test

^c^Comparison of the distribution of cases, among the categories of each factor, between implants in prosthesis with and without cantilever

^d^Pearson’s Chi-squared test

^e^Fisher’s exact test

^f^For the cases with available information

^g^It includes 8 implants in 3 former smokers

The total number of marginal bone level double measurements (mesial and distal sides of each implant) was 2,909, with 2,238 double measurements for implants supporting prostheses without cantilever and 671 for implants supporting prostheses with a cantilever.

The following tables show data on MBL distributed by different periods of follow-up, separated by implants in different locations within 2-implant-cantilevered prostheses (Table 2), 2-implant prostheses with an intermediary pontic (Table 3), and within 3-implant-supported prostheses (Table 4). There was a general slow and progressive increase in MBL over time, but with no statistically significant difference in the mean values between implants in different locations within the same type of prosthesis.

**Table 2:** Data on marginal bone loss distributed by implants in different locations within 2-implant-cantilevered prostheses

Follow-up^a^	*n*	Implant adjacent to cantilever	Implant distant from cantilever	*p* value^b^
		mean ± SD (min, max)		
0–1 year	110	− 0.17 ± 0.32 (− 1.39, 0.01)	− 0.22 ± 0.42 (− 1.59, 0.00)	0.592
1–2 years	36	− 0.70 ± 0.50 (− 1.66, 0.38)	− 0.61 ± 0.45 (− 1.61, 0.12)	0.407
2–3 years	22	− 0.87 ± 0.69 (− 2.89, 0.00)	− 0.77 ± 0.46 (− 2.22, 0.14)	0.925
3–4 years	18	− 0.99 ± 0.75 (− 2.55, 0.08)	− 1.00 ± 0.63 (− 2.39, 0.04)	0.743
4–5 years	24	− 0.94 ± 0.61 (− 2.42, 0.00)	− 1.07 ± 0.49 (− 1.98, − 0.11)	0.328
5–10 years	74	− 1.52 ± 0.83 (− 4.38, − 0.26)	− 1.52 ± 0.75 (− 3.76, − 0.41)	0.719
10–15 years	35	− 1.78 ± 0.83 (− 4.28, − 0.69)	− 1.93 ± 0.87 (− 3.30, 0.18)	0.772
15–30 years	30	− 2.19 ± 0.61 (− 3.59, − 1.18)	− 2.15 ± 0.73 (− 3.37, − 0.88)	0.804

Values in millimeters. Negative values correspond to bone loss

*SD* standard deviation

^a^Not all implants had radiological follow-up under all these follow-up periods, and some implants could have had more than one radiological follow-up under the same follow-up period

^b^Comparison of the mean values between implants in different positions; Mann–Whitney test

**Table 3:** Data on marginal bone loss distributed by implants in different locations within 2-implant prostheses with an intermediary pontic

Follow-up^a^	*n*	Mesial implant	Distal implant	*p* value ^b^
		mean ± SD (min, max)		
0–1 year	48	− 0.23 ± 029 (− 0.99, 0.00)	− 0.19 ± 0.28 (− 0.83, 0.30)	0.539
1–2 years	10	− 0.83 ± 0.89 (− 2.40, 0.03)	− 0.76 ± 0.68 (− 2.23, 0.14)	0.853
2–3 years	16	− 0.92 ± 0.88 (− 2.69, 0.14)	− 0.87 ± 1.05 (− 3.99, 0.00)	0.539
3–4 years	8	− 1.37 ± 1.01 (− 2.84, − 0.01)	− 1.08 ± 0.97 (− 2.41, 0.04)	0.505
4–5 years	5	− 0.75 ± 0.57 (− 1.55, − 0.01)	− 1.53 ± 1.41 (− 3.84, 0.00)	0.421
5–10 years	25	− 1.68 ± 1.16 (− 3.93, 0.00)	− 1.71 ± 1.29 (− 4.12, 0.05)	0.907
10–15 years	14	− 1.25 ± 0.40 (− 1.74, − 0.43)	− 1.40 ± 1.35 (− 5.11, 0.00)	0.734
15–30 years	21	− 1.42 ± 0.72 (− 2.49, − 0.46)	− 1.95 ± 1.23 (− 5.31, − 0.29)	0.089

Values in millimeters. Negative values correspond to bone loss

*SD* standard deviation

^a^Not all implants had radiological follow-up under all these follow-up periods, and some implants could have had more than one radiological follow-up under the same follow-up period

^b^Comparison of the mean values between implants in different positions; Mann–Whitney test

**Table 4:** Data on marginal bone loss distributed by implants in different locations within 3-implant-supported prostheses

Follow-up^a^	*n*	Mesial implant	Middle implant	Distal implant	*p* value^b^
		mean ± SD (min, max)			
0–1 year	297	− 0.16 ± 0.35 (− 1.57, 0.66)	− 0.17 ± 0.33 (− 1.29, 0.39)	− 0.13 ± 0.32 (− 2.15, 0.56)	0.600
1–2 years	63	− 0.58 ± 0.66 (− 3.54, 0.52)	− 0.71 ± 0.62 (− 2.47, 0.27)	− 0.59 ± 0.53 (− 2.35, 0.41)	0.301
2–3 years	32	− 0.53 ± 0.54 (− 2.09, 0.31)	− 0.71 ± 0.57 (− 2.72, 0.09)	− 0.56 ± 0.50 (− 1.67, 0.50)	0.345
3–4 years	51	− 0.89 ± 0.87 (− 4.83, 0.24)	− 0.94 ± 0.76 (− 3.28, 0.28)	− 0.87 ± 0.57 (− 2.69, 0.00)	0.842
4–5 years	37	− 1.11 ± 0.73 (− 2.69, 0.18)	− 1.26 ± 0.66 (− 3.36, − 0.21)	− 1.15 ± 0.59 (− 2.61, − 0.32)	0.665
5–10 years	115	− 1.19 ± 0.99 (− 5.25, 0.46)	− 1.21 ± 0.90 (− 3.66, 0.52)	− 1.16 ± 0.74 (− 3,31, 0.23)	0.913
10–15 years	72	− 1.36 ± 0.87 (− 3.30, 0.18)	− 1.65 ± 1.04 (− 4.14, − 0.05)	− 1.70 ± 0.84 (− 3.47, − 0.13)	0.124
15–30 years	72	− 1.70 ± 0.87 (− 4.94, 0.18)	− 1.87 ± 1.12 (− 5.71, − 0.29)	− 1.77 ± 0.90 (− 3.75, 0.00)	0.953

Values in millimeters. Negative values correspond to bone loss

*SD* standard deviation

^a^Not all implants had radiological follow-up under all these follow-up periods, and some implants could have had more than one radiological follow-up under the same follow-up period

^b^Comparison of the mean values between implants in different positions; Kruskal–Wallis test

**Fig. 3:**
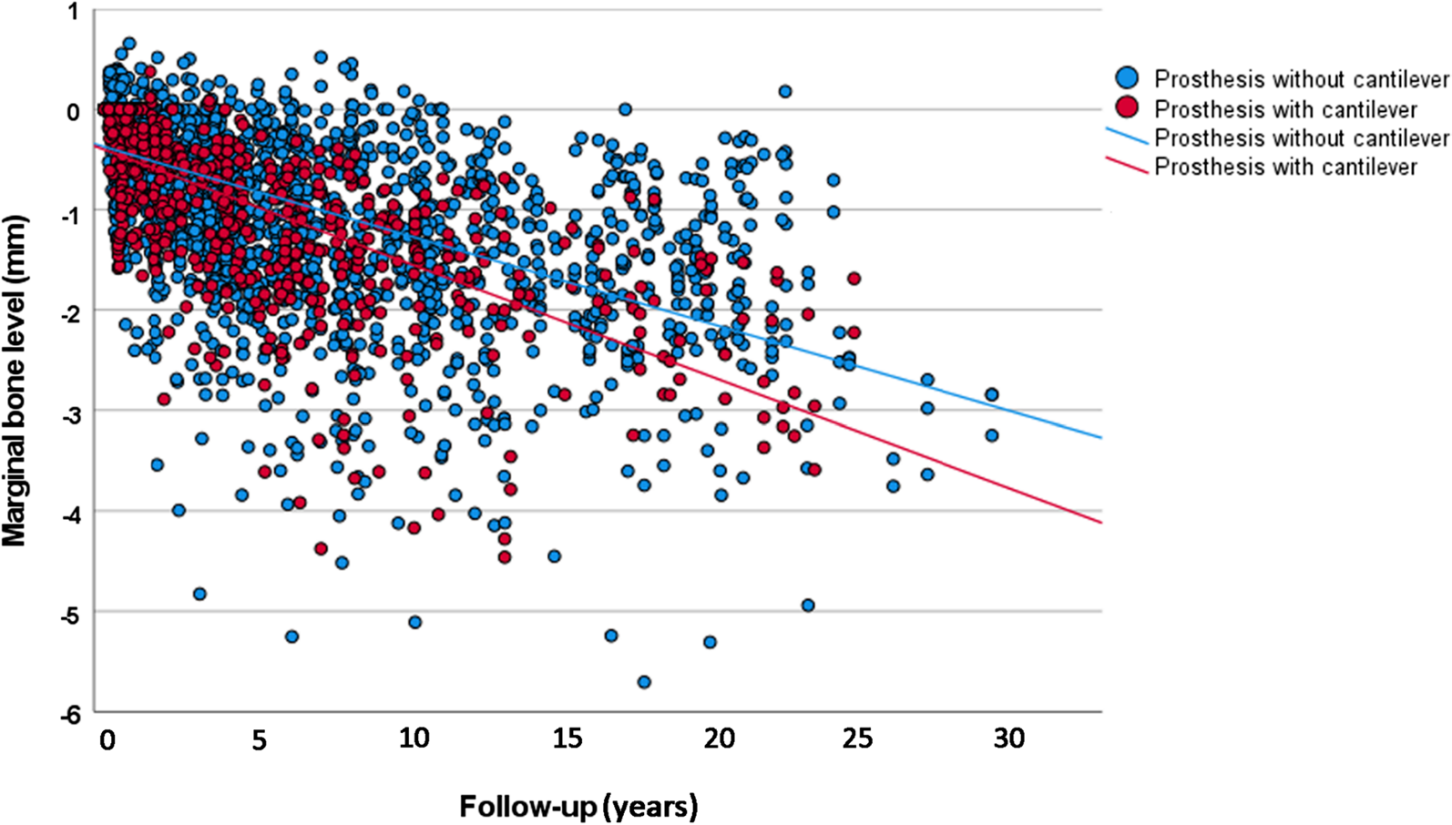
Scatter plot of the marginal bone level measurements in function of time of follow-up, for presence of absence of cantilever. Each dot represents one marginal bone level double measurement (mesial and distal sides of each implant)

The univariate linear regression analysis showed that the mean loss of marginal bone over time was statistically significantly different between the categories of the following variables (Table 5): cantilever, age, sex, implant surface, bone quantity, bone quality, and bruxism. The scatter plot with a comparison of MBL over time between prostheses with and without cantilever is presented (Fig. 3).

**Table 5:** Univariate linear regression analysis for MBL

Factor	Linear equation^a^	*p* value^b^	R^2^ linear
Cantilever			
No	y = − 0.37 − 0.00725x	0.003	0.337
Yes	y = − 0.40 − 0.00930x		0.473
Age (years)			
< 56	y = − 042 − 0.00735x		0.336
56.0–65.9	y = − 0.38 − 0.00692x	0.001	0.401
≥ 66	y = − 0.29 − 0.01000x		0.383
Sex			
Male	y = − 0.36 − 0.00626x	< 0.001	0.356
Female	y = − 0.39 − 0.00949x		0.409
Jaw			
Maxilla	y = − 0.35 − 0.00997x	0.978	0.408
Mandible	y = − 0.38 − 0.00700x		0.357
Jaw position			
Anterior	y = − 0.50 − 0.00815x	0.677	0.233
Posterior	y = − 0.38 − 0.00764x		0.370
Implant surface			
Turned	y = − 0.41 − 0.00702x	< 0.001	0.350
Modified	y = − 0.30 − 0.01000x		0.387
Implant diameter			
< 3.75 mm	y = − 0.27 − 0.01000x	0.996	0.370
3.75 mm	y = − 0.30 − 0.00811x		0.342
> 3.75 mm	y = − 0.30 − 0.00811x		0.551
Bone quantity			
A–B	y = − 0.35 − 0.00727x	0.002	0.355
C–D–E	y = − 0.43 − 0.00870x		0.394
Bone quality			
1–2	y = − 0.34 − 0.00747x	0.011	0.370
3–4	y = − 0.41 − 0.00776x		0.355
Smoking^c^			
No	y = − 0.44 − 0.00696x	0.451	0.350
Yes^d^	y = − 0.30 − 0.00933x		0.379
Bruxism^c^			
No	y = − 0.32 − 0.00682x	0.006	0.479
Yes	y = − 0.43 − 0.00745x		0.344
Diabetes			
No	y = − 0.40 − 0.00797x	0.739	0.371
Yes	y = − 0.47 − 0.00483x		0.298

^a^For the linear equation, “x” represents the number of months

^b^Comparison of the slope of the equation (variation of MBL in mm in time) between groups

^c^For the cases with available information

^d^It includes 8 implants in 3 former smokers

Most categories had a moderate degree of linear correlation (R^2^ linear) with MBL over time, with some presenting a weak degree of linear correlation, namely, absence of cantilever, age < 56 years, anterior region of the jaws, implants of 3.75 mm of diameter, absence of bruxism, and presence of diabetes.

**Fig. 4:**
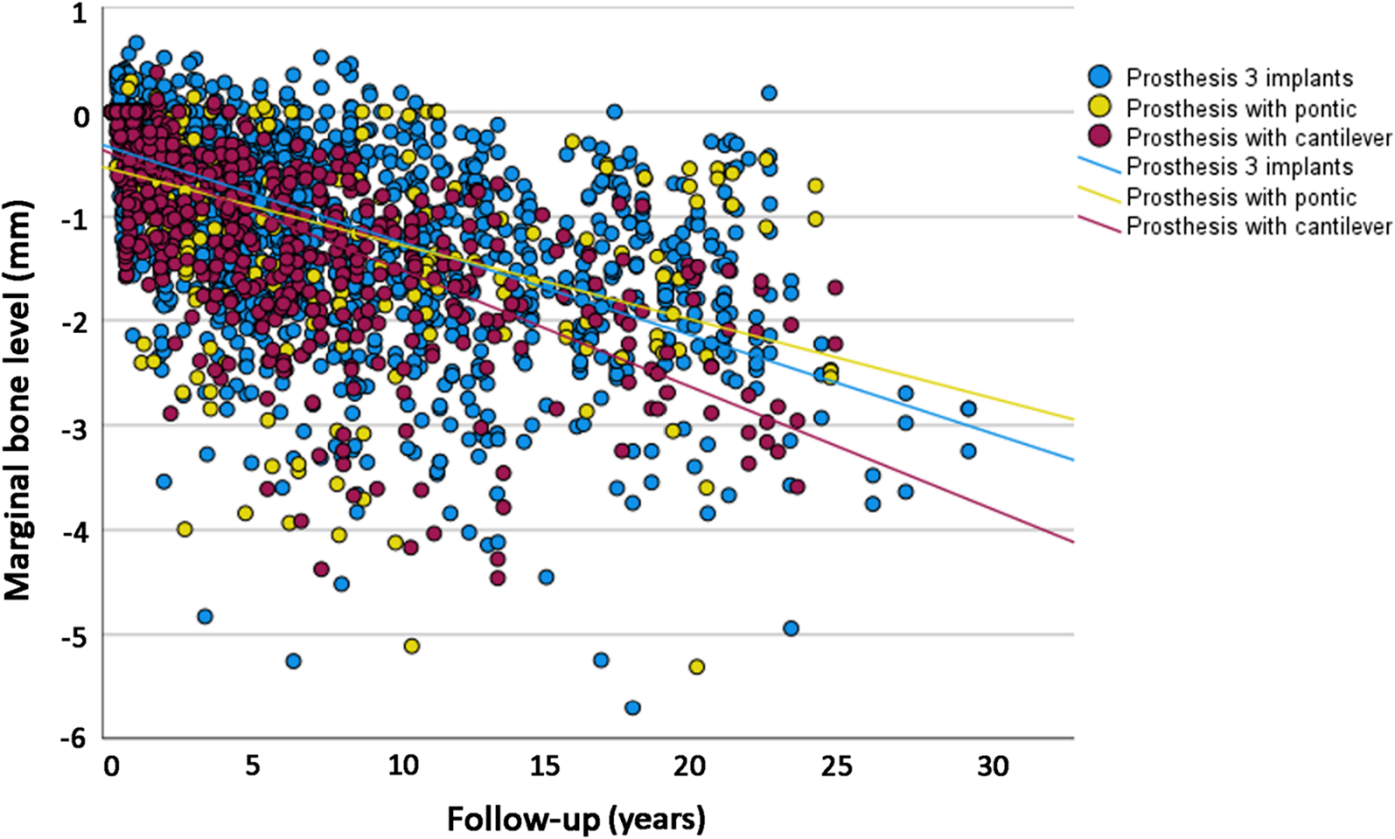
Scatter plot of the marginal bone level measurements in function of time of follow-up, for 3-unit prosthesis supported by two implants with a cantilever (purple line and dots), 3-unit prosthesis supported by two implants with a pontic (yellow line and dots), and 3-unit prosthesis supported by three implants (blue line and dots). Each dot represents one marginal bone level double measurement (mesial and distal sides of each implant)

A univariate linear regression sub-analysis for MBL was done comparing the three groups of prosthesis configuration, namely, 3-unit prosthesis supported by two implants with a cantilever, 3-unit prosthesis supported by two implants with a pontic, and 3-unit prosthesis supported by three implants (Table 6). The prosthesis with cantilever was still the one showing a higher estimated MBL over time among the groups. The scatter plot with a comparison of MBL over time between the three different prosthetic configurations is presented (Fig. 4).

**Table 6:** Univariate linear regression analysis for MBL

Factor	Linear equation^a^	*p* value^b^	R^2^ linear
Prosthesis configuration			
Three implants	y = − 0.34 − 0.00747x	< 0.001	0.366
Two implants with a pontic	y = − 0.55 − 0.00599x		0.208
Two implants with a cantilever	y = − 0.40 − 0.00930x		0.473

^a^For the linear equation, “x” represents the number of months

^b^Comparison of the slope of the equation (variation of MBL in mm in time) between groups

The results of the linear mixed-effects model (Table 7) suggested that the presence of cantilever, sex (worse for women), implant surface (worse for modified surface implants), bone quality (worse for poor bone qualities), and bruxism (worse for probable bruxers), had a statistically significant influence on MBL over time.

**Table 7:** Linear mixed-effects model for MBL over time

Predictor variables	F statistic	*p* value
Cantilever	45.032	< 0.001
Age	2.122	0.145
Sex	143.143	< 0.001
Implant surface	63.869	< 0.001
Bone quantity	0.050	0.824
Bone quality	10.083	0.002
Bruxism	40.013	< 0.001

## Discussion

The aim of this retrospective study was to investigate whether a cantilever prosthetic arm was associated with an increased MBL over time in patients rehabilitated with short implant-supported partial fixed prostheses. Based on present findings, it can be suggested that there is a statistically significant difference in the mean MBL over time with regard to cantilever. Other factors were also associated with this outcome, namely, patient’s sex, implant surface, bone quality, and bruxism.

The greater MBL over time observed in short prostheses with cantilever in comparison with prostheses without cantilever can be associated with the possible deleterious unequal transmission of forces from the prosthesis to the crestal bone when a cantilever arm is present, in agreement with many FEA and photoelastic studies on the subject [4, 6, 7, 20]. A FEA study that investigated the exact same of types of 3-unit prostheses as in the present study observed that the highest stress in bone around titanium implants was calculated in the model with prosthesis supported by two implants with a cantilever. Less stress was found in the model with a conventional FPD on two implants, and lowest stress was calculated in the model with three connected crowns supported by three implants [21]. A cantilever's amplified force may result in micromovements of the implant, which in turn is suggested to cause bone loss [1, 4, 5, 13]. It is, however, a matter of debate if the negative effect of cantilever observed in the present study may be clinically significant, due to the small difference of the estimated MBL over long periods of follow-up between the groups.

Women presented a statistically greater estimated MBL over time than males. It was not possible to find a reasonable explanation to this finding, but this may be related to factors not investigated in the present study [22], which might be associated with different patients in the present cohort. There is a very limited number of studies reporting data on MBL separated between men and women, and the reason for this difference was unknown.

The estimated greater MBL over time in implants with modified surface in comparison with implants with turned/machined surface may be due to the fact that rougher implant surfaces are more susceptible to accumulation of bacteria on hard surfaces [23–26]. A roughened surface increases the susceptibility for peri-implantitis, as well as reduces the treatment efficacy of the bacteria biofilm [27].

Greater MBL over time in implant sites of poorer bone quality could be related to the looser trabecular configuration and thinner cortical bone of this type of bone in relation to bones with higher density [28]. These anatomical features of poor-quality bone may negative impact clinical outcomes. Increased bone quality, meaning bone with higher density of trabecular and thick or thin cortical bone, has been associated with a decrease in bone loss [29]. Poor bone quality may result in not only in higher implant rates [30], but also in a frequent high loss of bone [31, 32].

The negative impact of bruxism on MBL over time could be associated the absence of a periodontal ligament around dental implants, which may limit the amount of feedback the that the central nervous system receives, which in turn cause a reduction in the tactile sensitivity around implants [33]. As a result, prostheses supported by implants are more likely to be subjected to higher loads during episodes of bruxism dues to the reduced tactile sensitivity [34–37]. The results of the first clinical study comparing MBL around implants in a group of bruxers in relation to a matched group of non-bruxers suggested that bruxism increases the risk of MBL over time [38].

This study is not without limitations. Its retrospective nature is associated with a lack of complete documentation in the patients’ records, since the study was not planned before the patients were treated. Data on many variables may have been not recorded in the dental chart. This issue may have been of higher impact when it comes to the periodontal history and status of the patients. Although patients with a history or with signs of periodontal disease were treated in a difference department of the aforementioned clinic, and, therefore, not included in the present study, this does not preclude that some of the patients may have developed peri-implantitis later. The variation in the follow-up time was another limitation. While most patients had a follow-up of 3 years or a bit more, others were followed up for more than 30 years.

## Conclusions

The use of a cantilever extension is suggested to negatively affect the bone marginal level over time around implants supporting 3-unit FPDs. However, due to the small difference of the estimated MBL over long periods of follow-up between the groups, it is a matter of debate if the observed negative effect of cantilever may be of clinical significance. Other factors are also suggested to influence MBL over time, namely, women, implant modified surface, poor bone quality, and bruxism.

## Data Availability

Restrictions apply to the availability of these data. Data were obtained from patients treated at Folktandvården Skåne AB, Malmö, Sweden, and cannot be shared, in accordance with the General Data Protection Regulation (EU) 2016/679.

## Abbreviations

FEA: Finite-element analysis
FPD: Fixed partial denture
MBL: Marginal bone loss
